# The effect of ubiquitination and deubiquitination to imatinib resistance in gastrointestinal stromal tumors

**DOI:** 10.3389/fonc.2025.1581920

**Published:** 2025-07-25

**Authors:** Huade Huo, Haolin Li, Xinlin Yang, Shu Wang, Yan Zhao, Jianjun Yang

**Affiliations:** ^1^ Department of Digestive Surgery, Xijing Hospital of Digestive Diseases, Fourth Military Medical University, Xi’an, China; ^2^ State Key Laboratory of Holistic Integrative Management of Gastrointestinal Cancers and National Clinical Research Center for Digestive Diseases, Xijing Hospital of Digestive Diseases, Fourth Military Medical University, Xi’an, China; ^3^ Faculty of Arts, University of Auckland, Auckland, New Zealand

**Keywords:** gastrointestinal stromal tumors, imatinib, drug resistance, ubiquitination modifications, deubiquitination modification

## Abstract

Gastrointestinal stromal tumor (GIST) is the most common mesenchymal tumor. Imatinib, as a receptor-type tyrosine kinase inhibitor (TKI), becomes a first-line drug for adjuvant therapy and prognosis. However, patients are facing with the problem of primary and secondary drug resistance when using imatinib, which affects the effect of imatinib. Thus, it is particularly important to explore the mechanism of drug resistance. Ubiquitination and deubiquitination process have been proofed to performance as posttranslational modifications (PTMs) to influence the occurrence and progression of most tumors. Hence, we attach importance to these mechanisms and found that GIST resistance may be related to ubiquitination and deubiquitination in regulating exosome secretion, autophagy, apoptosis and ferroptosis. Through clarifying these connections, this review aims to offers insights and hope for therapeutic advancements of imatinib-resistant GIST patients and the use of specific ubiquitin modifications as markers in the future.

## 1 Introduction

GISTs is a type of tumor that originates from the stromal cells of Cajal. The most common driver mutations in GISTs occur in kinase insert domain receptor (KIT) (60-70%) and platelet—derived growth factor receptor alpha (PDGFRA) (10-15%) (1). In the case of KIT, the binding of KIT ligands and stem cell factors (SCFs) to the extracellular domain of the receptor leads to its dimerization and activation of the intracellular tyrosine kinase domain and receptor through autophosphorylation of specific tyrosine residues (2). As for PDGFRA, activation of this type is intrinsically driven by acquired mutations causing conformational changes. Especially for mutations which cluster in critical domains (exons 12/14/18) that disrupt auto-inhibition and stabilize active states (1).

Currently, surgical resection is the most common treatment for resectable GISTs, and about 60% of patients can be cured by surgery (3). However, for patients with advanced metastatic GISTs and locally advanced unresectable GISTs, imatinib plays an important role. Imatinib was originally designed for breakpoint cluster region-Abelson murine leukemia viral oncogene (BCR-ABL) translocation in chronic myeloid leukemia and has subsequently shown to be effective against KIT and PDGFRA tyrosine kinases in GIST (4).Although more than 80% of patients with GIST can benefit from imatinib, many patients develop imatinib resistance after treatment. Universally acknowledged mechanisms include: decreased drug consuming, metabolism and degradation of drugs, evasion of apoptosis, mutations in the drug target proteins (5). Resistance to imatinib in GIST patients can be divided into primary resistance and secondary resistance. Primary drug resistance is that GIST has no effect on imatinib at the beginning, which is mainly related to GIST genotype. For example: the mutation of PDGFRA exon 18 D842V can mediate primary imatinib resistance (6). About 50% advanced GISTs developed tumor progression after the initial efficacy of imatinib after 2 years of medication which is defined as secondary resistance (7). Ubiquitination is an important posttranslational modification (PTMs) in eukaryotes that begins with the attachment of a single ubiquitin molecule to a substrate lysine residue to mediate biochemical reactions such as organelle recognition and protein degradation (8). In contrast, deubiquitination is mediated by a family of deubiquitinating enzymes (DUBs), which specifically recognize ubiquitin chains for deubiquitination (9, 10). This review focuses on the current status of treatment of gastrointestinal stromal tumors, and sorts out the relationship between ubiquitination or deubiquitination modifications and GIST progression as well as imatinib resistance.

## 2 Current treatment of gastrointestinal stromal tumors

### 2.1 Surgical management strategies for GISTs

From a surgical perspective, the goal of resection is to ensure surgical margins are negative and prevent the rupture of tumors to avoid recurrence (11, 12). For patients with large tumors (>5 cm), those with invasion of adjacent organs, or metastatic patients, tyrosine kinase inhibitor (TKI) targeted therapy (such as imatinib) is preferred over immediate surgery. Proper surgical treatments can be applied after reaching the maximum response at 6 to 12 months (1). For micro/small GISTs (<2 cm), endoscopic ultrasound surveillance (annually) is recommended for gastric/duodenal lesions, while rectal lesions mandate resection regardless of size. In wild-type GISTs, SDH-deficient cases require resection of visible lesions with frequent lymph node dissection, whereas NF1-associated GISTs, given their indolent biology, only require surgery for symptomatic lesions without radical intent (3). Notably, all postoperative intermediate-to-high-risk GIST patients—excluding those harboring the PDGFRA D842V mutation—should undergo prolonged adjuvant TKI therapy (imatinib 400 mg/day for 3 years).

### 2.2 Pharmacological interventions for GISTs

Patients with metastatic disease should not be operated on earlier but being treated with TKIs first. Over the past 20 years, TKIs have been recognized as the preferred first-line treatment based on a series of clinical trials. Additionally, several active therapies corresponding to different symptoms have been identified, and imatinib as the main drug have been developed (1). Acting as TKIs, imatinib revokes the KIT signaling mainly through binding onto the ATP-binding site. Prior to imatinib treatment, 50 percent of patients who underwent surgical resection of GIST relapsed within five years, with a 50 percent five-year survival rate (13, 14). Additionally, the partiality of imatinib to this site depends on the mutation of receptor, which explains why imatinib improves prognosis and survival outcomes, but rarely directly cured due to the emergence of resistant cells within the tumor (15). However, the molecular mechanisms of imatinib resistance have not been elucidated. Despite from playing a significant role in the prognostic level of patients and controlling the progression of the disease, imatinib appears to be feasible and safe when used during preoperative treatment, as it does not lead to an increase in postoperative complications (16). Other TKIs used in treating GISTs includes sunitinib (a second-line drug against KIT exon 9 mutations (17)) and regorafenib (demonstrate significant efficacy in GISTs which had progressed after failure of both imatinib and sunitinib (18)).

### 2.3 Summary of changes in the way of managing GISTs

The timeline highlights pivotal transitions from surgical monotherapy (1980–2000) to molecularly targeted strategies (**Table 1**). Key milestones begin with the introduction of imatinib (2001–2002), which revolutionized metastatic GIST treatment (19). This was followed by the sequential approval of tyrosine kinase inhibitors for resistant disease—sunitinib in 2006 (20) and regorafenib in 2014 (21). Concurrently, optimization of adjuvant imatinib duration evolved from 1-year (2009) to 3-year regimens (2012), significantly improving survival in high-risk resected GIST (22, 23). The precision therapy era emerged in 2020 with mutation-specific agents, including avapritinib for PDGFRA D842V-mutant tumors and ripretinib for ≥fourth-line therapy (24). Further advancing individualized management, molecular subtype-directed approaches such as larotrectinib for NTRK fusion-positive GIST demonstrate targeted efficacy (25). Collectively, these advances transformed survival outcomes from a median of 10–20 months in the surgery era to multi-year survival with contemporary targeted regimens.

**Table 1:** Chronological evolution of therapeutic paradigms in GISTs management.

Timeline	Phase of therapy	Key advances	Refs
1980–2000	Surgery Era	Surgery as the only curative approach; median survival: 10–20 months	(1)
2001–2002	Targeted Therapy Breakthrough	Imatinib approved for advanced/metastatic GIST (first targeted agent)	(19)
2006	Second-Line Therapy	Sunitinib approved for imatinib-resistant GIST	(20)
2009	Adjuvant Therapy Established	1-year imatinib adjuvant therapy significantly reduced postoperative recurrence	(22, 23)
2012	Optimized Adjuvant Therapy	3-year imatinib for high-risk patients improved OS by 10% (SSG XVIII/AIO trial)	(22, 23)
2014	Third-Line Therapy	Regorafenib approved for imatinib/sunitinib-resistant GIST	(21)
2020	Precision Therapy	Avapritinib: Approved for PDGFRA D842V-mutant GIST; Ripretinib: Approved for ≥4th-line therapy	(24)
Now	Individualized Strategy	Larotrectinib (ORR: 75%)	(25)

## 3 GISTs disease progression with ubiquitination and deubiquitination modifications

Previous studies have found the unique and definitive role that ubiquitin0061tion and deubiquitination have attended in the progression of many diseases as well as tumors. It is well acknowledged that through competing ubiquitin conjugation and deubiquitination that controls both proteasomal degradation and signaling complex formation, this system can control many classic pathways, for example TNF signaling pathway (26). In this way, it participates in various disease progression thus arising huge concern on targeting specific proteins working with UB molecule to seek for a better therapy. Among E1, E2, E3 and DUBs, the DUBs appear to be more misregulated in many tumors and play critical roles in tumorigenesis as well as progression (27). Among tumors, osteosarcoma who origins from mesenchymal tissues have been proofed to relate tightly with E3 ligases, containing high amounts of cellular processes and signaling pathways (28). Additionally, UCHL1, part of the DUBs, can promote osteosarcoma cell proliferation and invasion while leading to the development of other mesenchymal tumors like uterine leiomyoma (29, 30). There have already been varieties of treatments targeting ubiquitination and deubiquitination modifications (**Table 2**). Such as PARP inhibitors (PARPi) in germline BRCA mutated (gBRCAm) breast cancer (31). Combined with chemotherapy or immunotherapy special protein inhibitors have received good results on improving overall survival rate while kind of avoiding facing the stage of TKI resistance of lung cancer patients (32). When it comes to the progression of GISTs, ubiquitination and deubiquitination modifications can control apoptotic and ferroptosis through different proteins and cause GIST to develop and deteriorate.

**Table 2:** Dysregulation of ubiquitination/deubiquitination in major cancer types and therapeutic implications.

Cancer type	Ubiquitination dysregulation	Deubiquitination dysregulation	Clinical evidence/examples	Refs
Breast Cancer	BRCA1 mutations	USP7 amplifications	PARPi FDA-approved for BRCA-mutated BC	(31)
Prostate Cancer	MDM2 amplification	USP22 overexpression	MDM2 inhibitors (Idasanutlin) DUB inhibitors targeting USP22	(83, 84)
Colorectal Cancer	FBXW7 mutations	OTUB1 overexpression	USP7 inhibitors with chemotherapy OTUB1 inhibitors	(85, 86)
Lung Cancer (NSCLC)	KEAP1 mutations	USP14 overexpression	USP14 inhibitors (b-AP15) with chemotherapy or immunotherapy	(32)
Leukemia (AML)	c-KIT mutations	USP7 activation	FT-827 (USP7 inhibitor) with Venetoclax	(87)

### 3.1 General mechanisms of ubiquitination and deubiquitination modifications

Ubiquitination is the covalent attachment of ubiquitin as a small molecule protein modifier to substrate proteins, which is involved in almost all cellular processes by mediating the degradation of proteins. The ubiquitin molecule is characterized by seven lysine residues that can continue to be used to link the ubiquitin molecule or to phosphorylation and acetylation, and to deliver more complex intracellular signals through the modification of the ubiquitin molecule (33). During ubiquitination process, ubiquitin molecules (Ub) are expressed as head-tail fusions with ribosomal proteins (RPs), which are then processed into free Ub by deubiquitinating enzyme (DUBs), exposing their characteristic diglycine C-terminus.

**Figure 1:**
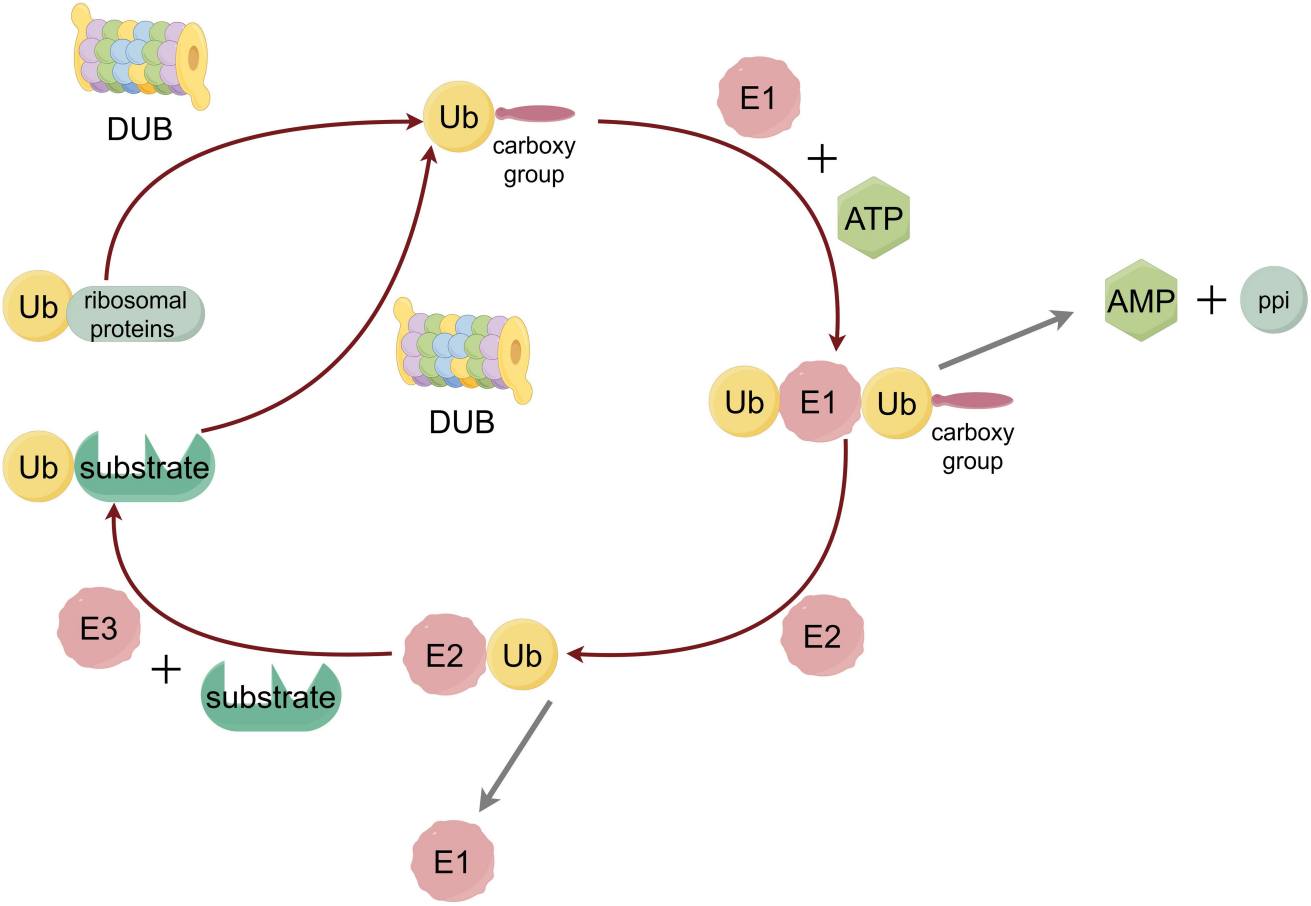
The process of ubiquitination. Ubiquitin protein is adenylytized at the C-terminal by E1 ubiquitin-activating enzyme, and then transferred from the cysteine residue at the active site of the E2 ubiquitin-conjugated enzyme to the lysine residue of the substrate protein, which is linked to the isopeptide bond between the lysine residue in the substrate protein by the action of E3 ubiquitin ligase.

Subsequently, the ubiquitin protein is processed by E1 ubiquitin-activating enzyme, E2 ubiquitin-conjugated enzyme and E3 ubiquitin ligase to be gradually transferred from E1 to the target protein (**Figure 1**) (34). At the same time, the E4 molecule, a persistent synthesis factor that is itself E3 but has activity that helps and shapes the formation of the chain can synergize with another E3 molecule to promote the formation of the Ub chain (35).

The process of deubiquitination of proteins is mediated by DUBs, which represent a large class of proteases that are specific for Ub, Ub conjugates, and Ub chains (36). There are five main types of deubiquitinating enzymes, namely: JAMM (JAB 1/MPN/Mov 34) domain DUB, UCH (Ub C-terminal hydrolase), USP (Ub-specific protease), OTU (ovarian tumor associated proteinase) and Josephin domain DUB (34). Inside the cell, the ubiquitination system regulates and participates in numerous biochemical reactions. Short-lived and soluble misfolded/unfolded proteins can be targeted and eliminated by the ubiquitin proteasome system (37). Acting as the key to the dynamic regulation of programmed cell death, ubiquitination can modulate autophagy. Such as reversible ubiquitination of core autophagy-inducible factors as subunits of the ULK1 and PI3K complexes, and has shown to be a common mechanism which turns on and off the autophagy process (38). Through participate into programmed cell death ubiquitination and deubiquitination may affect the TKI resistance which play roles by controlling certain mechanisms.

### 3.2 Elevated ubiquitination level of pro-apoptotic protein BIM in GIST suggests that it can affect disease progression

**Figure 2:**
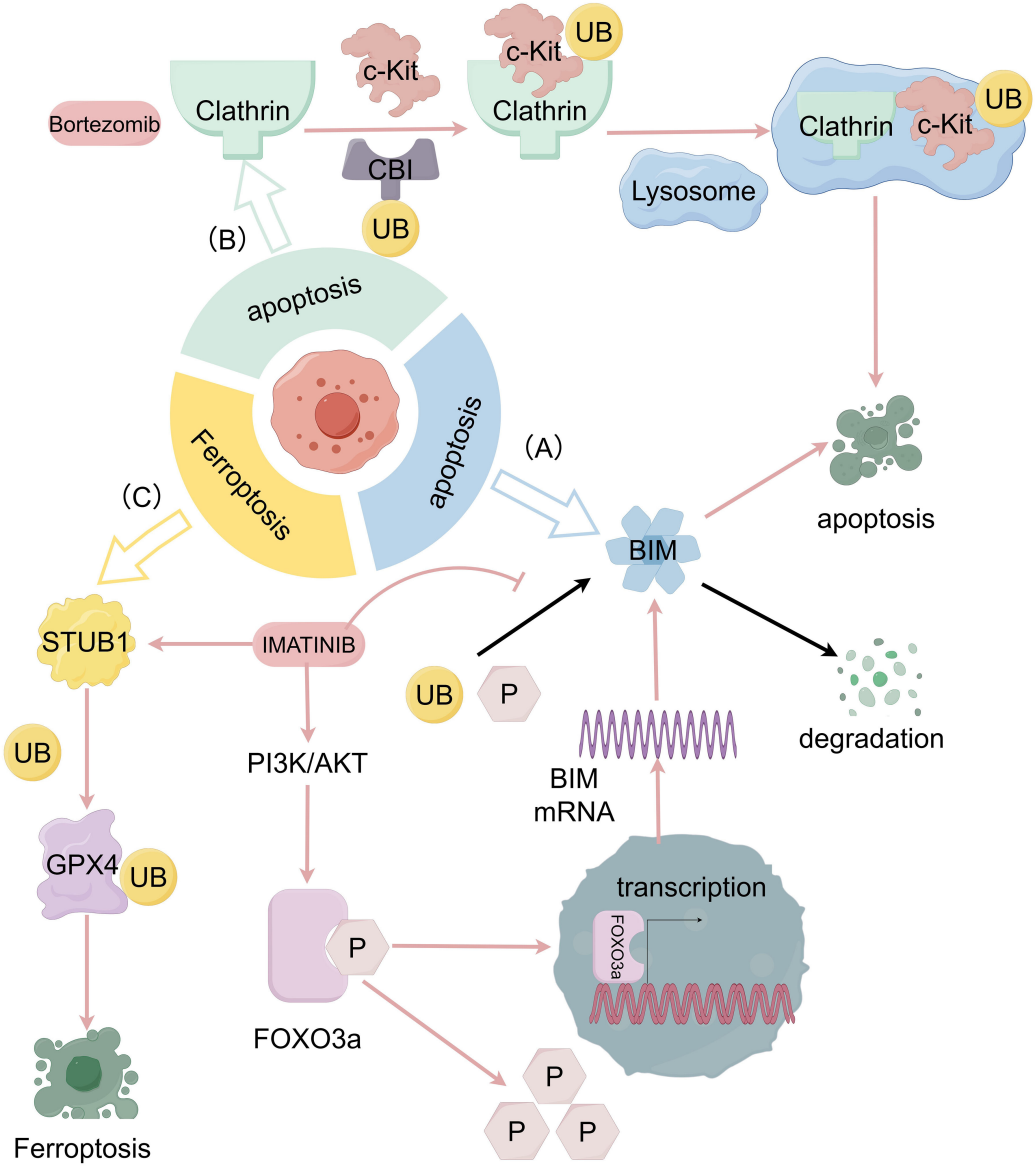
The relationship between GIST progression and ubiquitination and deubiquitination system. **(A)** Imatinib induces BIM transcription through the PI3K-AKT-FOXO 3a pathway and the ubiquitination and phosphorylation levels of BIM are also reduced through MAPK signaling pathway which prevent the degradation of BIM then cause the apoptosis of tumor cells. **(B)** With internalization of c-KIT engulfed by clathrin, Cbl can modify c-KIT which result in the c-KIT degradation in lysosomes and causes apoptosis of tumor cells as well. **(C)** Imatinib, in GIST can promote ubiquitination of the K191 site of GPX 4 by promoting the expression of STIP1 homology and u-box containing protein 1 (STUB 1), leading to degradation of GPX 4 protein and inducing ferroptosis.

In most GISTs, c-KIT receptor tyrosine kinase is carcinogenic and being constitutively activated (1). Within this type of GISTs, tumors can evade apoptosis by upregulating the ubiquitination and phosphorylation levels of bcl-2 interacting mediator of cell death (BIM) through transcriptional and post-translational mechanisms, which can lead to its degradation. Studies have shown that after imatinib treatment with the GIST 882 cell line, imatinib induces BIM transcription, while the mitogen-Activated Protein Kinase (MAPK) signaling pathway reduces the ubiquitination and phosphorylation levels of BIM through post-translational levels. The effect of imatinib ultimately leads to a rapid and sustained upregulation of the expression of the BIM molecule (**Figure 2A**), while other apoptotic factors do not show significant perturbation (39). This suggests that BIM upregulation can be used to trigger apoptosis through alternative therapies that inhibit c-KIT signaling. Such as frapine (40) which inhibits c-KIT transcription, and the heat shock protein 90 (HSP 90) inhibitor that targets c-KIT protein stability.

### 3.3 E3 ubiquitin ligase Cbl induces apoptosis in GIST cells by ubiquitination and degradation of internalized and engulfed c-KIT

The ubiquitin molecule with E3 ubiquitin-protein ligase and its mediated lysosomal pathways are generally involved in the degradation of membrane receptor proteins in the cell body (41). The proteasome inhibitor bortezomib (BOR) has shown to modulate the c-KIT-associated apoptosis cascade in leukemia cells by directly inducing c-KIT internalization and lysosome-induced degradation (42). This mechanism is also effective in GIST cells. By using dynasore (DY), an initiator protein inhibitor, to interfere with the formation of clathrin-mediated internalized coated vesicles, Ying Dong et al. demonstrated that KIT can be engulfed by clathrin-mediated internalization, followed by c-KIT degradation in lysosomes as a target protein modified by casitas B lymphoma-b (Cbl) (**Figure 2B**). PDGFRβ-dependent cell cycle arrest achieved with dasatinib and c-KIT internalization facilitated by bortezomib can be used in a coordinated combination to efficiently induce apoptosis in GIST cells (43).

### 3.4 Ubiquitination of GPX 4 inhabits the growth of tumor through inducing ferroptosis in gastrointestinal stromal tumors

Ferroptosis is an iron-dependent, non-apoptotic form of regulated cell death driven by lipid peroxidation (44). The main mechanism of ferroptosis lies on the inability to detoxify lipid hydroperoxides that leads to membrane rupture and cell death (45). Specifically, glutathione peroxidase 4 (GPX 4) is the only cellular enzyme capable of reducing lipid peroxides to lipids and has been used as a target for various ferroptosis inducers (46). Xiangfei Sun et al. found that imatinib in GISTs can promote ubiquitination of GPX 4, leading to degradation of GPX 4 protein and inducing ferroptosis (47) (**Figure 2C**). Among them, RAS-selective lethal 3 (RSL3)is an FDA-approved GPX 4-specific inhibitor that has shown to inhibit the growth, invasion, and metastasis of a variety of tumors (48). The combination of RSL3 and imatinib may also become a new therapeutic strategy.

## 4 Imatinib resistance with ubiquitination and deubiquitination of GISTs

As mentioned previously, the mechanism of GISTs imatinib resistance is quite complex, which in general can be divided into primary and secondary resistance. Primary resistance refers to the progression of tumors during the first six months of treatment, mainly seen in KIT exon 9 mutant GIST and PDGFRA exon 18 D842 V mutant GIST treated with 400 mg of imatinib per day, as well as wild-type (mainly SDH-deficient) GIST, accounting for 10%-14%. Secondary mutation acquired during treatment is secondary resistance, accounting for 40%-50% (49, 50). For primary resistance, mutations in KIT exon 9 lead to receptor dimerization, which may hinder the binding of tyrosine kinase receptors to imatinib (51). Acquired cis-mutations in the ATP-binding domain (encoded by exon 13 or 14 of KIT and exon 14 of PDGFRA) or activation loop (encoded by exon 17 of KIT and exon 18 of PDGFRA) are the main causes of secondary resistance to KIT-mutant and PDGFRA-mutant GIST (52).

For tumor cells with different exon mutations, even if the binding strength of imatinib and KIT receptors is excluded, differences in signaling pathways of their downstream effects are also related to imatinib resistance, resulting in very different therapeutic effects and drug resistance (53, 54). In the progression of GIST, downstream signal transduction pathways of KIT molecule include MAPK, phosphatidylinositol 3’-kinase (PI3K), and Janus kinase/signal transducer and activator of transcription (JAK/STAT) pathways. Activation of KIT and PDGFRA mutations results in ligand-independent dimerization, constitutive activation, and subsequent uncontrolled intracellular signaling and cell growth (55, 56). Among them, PI3K/AKT/mTOR pathways have been shown to play an important role in KIT signaling in GIST resistance (57). Bosbach et al. found that in a mouse model the inactivate of PI3K kinase binding site pY719 had a longer survival and did not develop GIST, suggesting this locus may recruit PI3K upon phosphorylation, and that GIST progression in mice can be reversed by inactivating it (58). Through a review of existing studies, we found that ubiquitination modification also plays an important role in GIST imatinib resistance by affecting the transmission of downstream signaling pathways, and may affect further adjuvant therapy of GIST by becoming a target of action drugs and tumor markers.

### 4.1 Ubiquitination system regulates exosome secretion leading to the transmission of imatinib resistance

**Figure 3:**
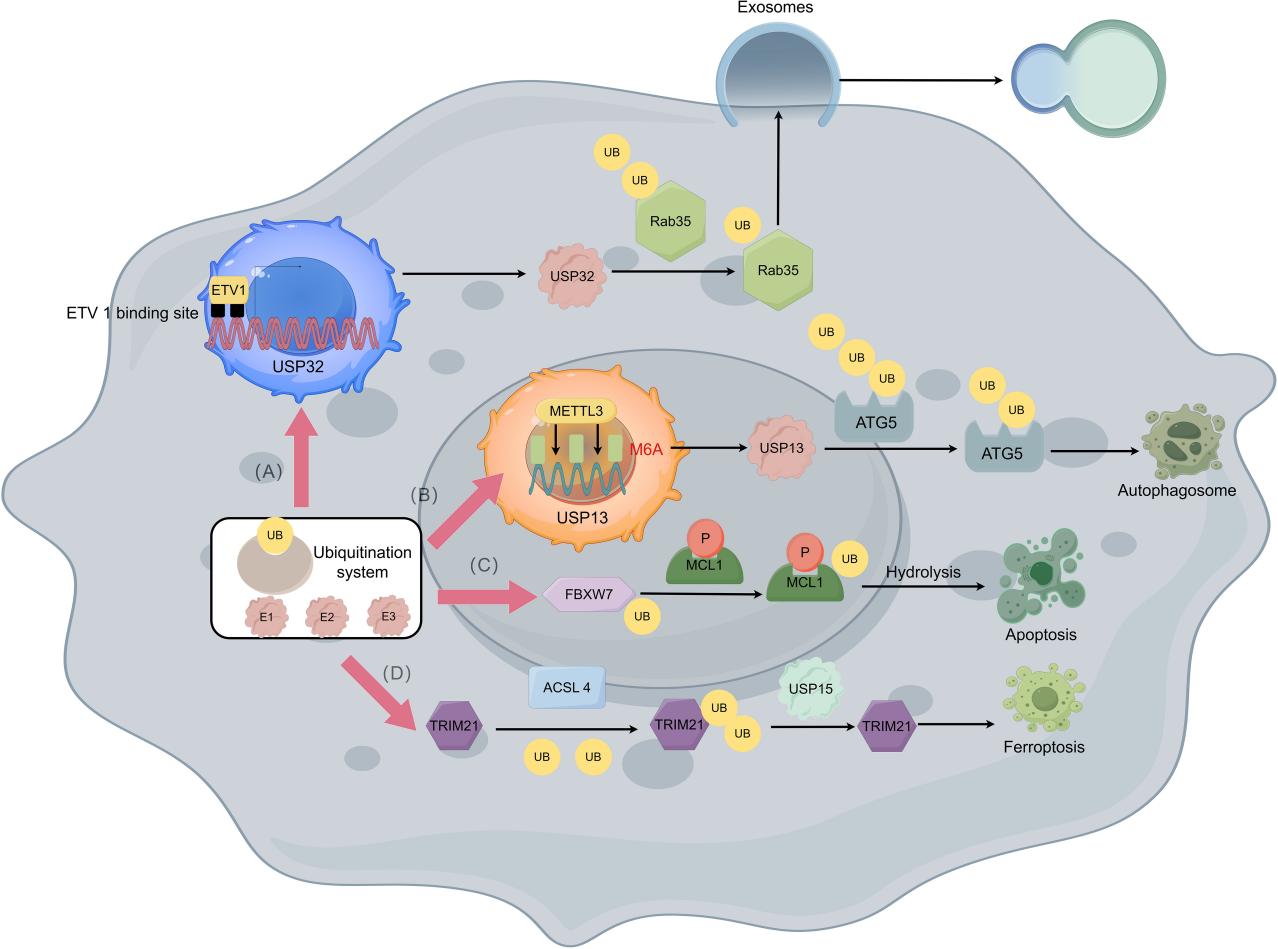
Imatinib resistance with the ubiquitination and deubiquitination of GISTs. **(A)** ETV1 can bind to two potential ETV 1 binding site upstream the USP32 promoter thus promoting polycystic transport by regulating the expression and localization of Rab35 through USP32 which can reduce the polyubiquitination of Rab35. With the help of Rab35, exosome secretion in GIST cells is upregulated which causes the resistance of imatinib in GIST cells to be transmitted. **(B)** Insulin like growth factor 2 mRNA binding protein 2 (IGF2BP2) can read METTL3-mediated m6A modification thus stabilizing USP13 mRNA, while USP13 can work as a deubiquitination protein that deubiquitinate UB molecules attached to ATG5 and causes protective autophagy of GIST cells. **(C)** FBXW7 can target the phosphorylated MCL1 molecule which leads to the degradation of MCL1, thereby relieving the inhibition of apoptosis and showing increased sensitivity to imatinib. **(D)** TRIM21 promotes the degradation of ACSL 4 by using a K48-linked polyubiquitin chain to lead to drug resistance, while ubiquitin-specific protease 15 (USP 15) upregulates the stability of ACSL 4 molecules through its deubiquitination activity and promotes ferroptosis in GIST cells to against resistance.

In normal humans, the Ras-associated protein (Rab) controls vesicle trafficking by promoting organelle dynamics and the fusion of vesicles with receptor membranes (59). In GISTs, the Ras-related protein Rab-35 (RAB35) is also involved in regulating exosome secretion, thereby transporting special membrane pieces with imatinib resistance mutations through the GIST cells (60). In general, RAB35 is regulated by degradation in a ubiquitin-β proteasome system dependent manner (61), and further analysis of the type of demulti-ubiquitination on RAB35 protein shows that USP 32 (ubiquitin-specific protease 32) can effectively reduce the polyubiquitination of Lys 48 (K48) linkage of RAB35, but has no significant effect on the non-degradable Lys 63 (K63) linked polyubiquitination of RAB35. Finally, ETS variant transcription factor 1 (ETV 1), as a lineage-specific survival factor, can promote polycystic transport by regulating the expression and localization of RAB35 by USP32, thereby upregulating exosome secretion in GIST cells (**Figure 3A**) (60). As a result, exosomes secreted by imatinib resistance cells can enhance the ability for imatinib sensitive cells to resist imatinib (60, 62). Additionally, the severity of resistance transmission can depend on the amounts of exosomes and internalization by GISTs cells.

### 4.2 Ubiquitination system regulates autophagy and imatinib resistance

USP 13 can affect imatinib resistance in GISTs by modulating the stability of autophagy-associated protein 5 (ATG5). USP 13 is an important member of the USP ubiquitin-specific processing enzyme subfamily, which controls the ubiquitination state of different substrates involved in multiple processes, thereby regulating cell cycle, autophagy, and metabolism (63, 64). This molecule and ATG5 are highly expressed in IM-resistant GIST cell lines, and USP13 has been found to potentially stabilize ATG5 by removing the K48-linked polyubiquitin chain at residue K5 (65). METTL3-mediated m6A modification maintains USP13 expression (66) thereby increasing the degree of imatinib resistance (67) (**Figure 3B**). By regulating the above links in the mechanism of action, the degree of protective autophagy of GIST cells can be attenuated by reducing the expression or effect of USP13, and the combination of imatinib on this basis may lead to better therapeutic effects.

### 4.3 Ubiquitination system regulates the cell cycle and imatinib resistance

F-box and WD repeat domain-containing 7 (FBXW7) can enhance the sensitivity of GIST-T1 cells to imatinib through inhibition of MCL1 (68). FBXW7 is a key tumor suppressor and cell cycle regulator, and proteasomal degradation is triggered in human cells through ubiquitination of proteins (69). Xiyu Wu et al. (68) found in GIST-T1 cells that MCL1 is involved in regulating the sensitivity of GIST-T1 cells to imatinib by inhibiting apoptosis. More specifically, FBXW7 can target the phosphorylated MCL1 molecule and perform ubiquitination modification, which leads to the degradation of MCL1, thereby relieving the inhibition of apoptosis and showing increased sensitivity to imatinib (**Figure 3C**).

In addition, similar studies suggest that bortezomib may also improve imatinib resistance through cell cycle regulation. Previous studies have shown that bortezomib induces KIT internalization and degradation by binding KIT to the ubiquitin protein ligase casitas B-cell lymphoma protein (CBL) in KIT-dependent GIST cells, thereby inducing apoptosis in GIST cells (43). The expression of cyclin D1 and the activity of Hippo/YAP signaling pathway were significantly increased in KIT-independent GIST cells (70).

### 4.4 Ubiquitination system regulates ferroptosis and imatinib resistance

As mentioned earlier, ferroptosis plays an important role in GIST disease progression, and ferroptosis has shown to play a key role in GIST resistance as well (71, 72). Acyl-CoA synthase 4 (ACSL 4), as a key enzyme in inducing ferroptosis can regulate lipid biosynthesis (73). By using the GPX4 inhibitor RSL3 as an inducer of ferroptosis, Zhiwei Cui et al. found that ACSL 4 expression was upregulated and GIST resistance was inhibited after the use of RSL3 in the GIST-T1 and GIST-882 cell lines (74). Further, in GIST-resistant cells, tripartite-motif protein 21 (TRIM21) promotes the degradation of ACSL 4. On the contrary, USP 15 can upregulate the stability of ACSL 4 molecules to promote ferroptosis in GIST cells (**Figure 3D**), thereby reducing drug resistance (74). Therefore, the activity of ACSL 4 molecule and its mediated ferroptosis show its potential as a future therapeutic target for GIST, whether by inhibiting the ubiquitination of TRIM21 or promoting the deubiquitination of USP 15.

## 5 Discussion

The existing mainstream treatment methods for gastrointestinal stromal tumors include conventional surgery and targeted drug therapy, among which tyrosine kinase inhibitors represented by imatinib are the most commonly used treatment strategies (1). Although new therapeutic ideas, have shown some therapeutic efficacy (75–78), the current focus of clinical and scientific research is still to deal with the drug resistance problem caused by the long-term use of imatinib. Imatinib has shown to be effective against KIT and PDGFRA tyrosine kinases in GISTs, and patients treated with imatinib are associated with primary resistance and secondary resistance. In exploring the treatment of GISTs, we found that ubiquitination and deubiquitination modifications play an important role in GIST progression and drug resistance by influencing the transmission of downstream signaling pathways.

In general, ubiquitination and deubiquitination, as important modification methods, have critical significance for the stability of protein molecules at the post-transcriptional level, and this mechanism can also regulate cell translation and transcription by affecting the stability of specific molecules. In gastrointestinal stromal tumors (GIST), dysregulated ubiquitination drives pathogenesis: Elevated ubiquitination of pro-apoptotic protein BIM promotes its degradation, enabling apoptosis evasion. Imatinib counteracts this by suppressing MAPK signaling, reducing BIM ubiquitination and restoring its pro-apoptotic function. E3 ligase Cbl ubiquitinates internalized c-KIT receptors (activated oncogenic drivers in GIST), targeting them for lysosomal degradation. Combining proteasome inhibitor bortezomib with dasatinib enhances c-KIT degradation and apoptosis. Imatinib-induced GPX4 ubiquitination triggers ferroptosis (iron-dependent cell death via lipid peroxide accumulation) by degrading this key antioxidant enzyme. GPX4 inhibitors (RSL3) synergize with imatinib to amplify ferroptosis death. Collectively, targeting ubiquitination pathways (BIM stabilization, Cbl-mediated c-KIT degradation, and GPX4-driven ferroptosis) reveals promising therapeutic strategies against GIST.

Considering imatinib resistance, apoptosis and ferroptosis and abnormal autophagy cause the generation of imatinib resistance in GIST cells, and the regulation of these three mechanisms is expected to affect the drug resistance caused by mutations. Unlike apoptosis and ferroptosis, the inhibition of protective autophagy in GIST cells corresponds to a certain degree of controlling of drug resistance, while the promotion of apoptosis and ferroptosis through ubiquitination and deubiquitination pathways can reverse the increase of imatinib resistance in tumor cells caused by mutations (79, 80).

The intricate regulation of imatinib resistance in GISTs by ubiquitination pathways highlights several promising clinical strategies. Targeting USP32 or ETV1 could disrupt resistance-conferring exosome secretion, limiting its spread. USP13 inhibitors offer a direct route to block protective autophagy by destabilizing ATG5, sensitizing resistant cells. Enhancing FBXW7 activity or mimicking its effect could degrade MCL1, restoring apoptosis and imatinib sensitivity. Promoting ferroptosis by inhibiting TRIM21-mediated degradation or activating USP15 to stabilize ACSL4 represents a novel approach to kill resistant cells (74, 81). Critically, combining imatinib with agents targeting these specific ubiquitination nodes (deubiquitinase inhibitors, ferroptosis inducers like RSL3, or FBXW7 modulators) is a rational, multi-pronged strategy to overcome resistance and improve GIST treatment outcomes (68, 71, 82). It should be noted here that Ras-associated protein-mediated exosome secretion promotes the transmission of drug resistance between GIST cells, resulting in stubborn drug resistance that is more difficult to treat with (60). Considering a variety of benefit, this system still has lots to overcome to finally serve patients. Ubiquitin-related enzymes (USP32, USP13, TRIM21) typically regulate multiple substrates. Targeted inhibition may disrupt normal cellular functions, leading to unpredictable toxicity. Additionally, Ubiquitination pathways could intersect with other resistance mechanisms (BCL6-p53 axis). Isolated targeting of ubiquitination may be compromised by bypass signaling (p53 inactivation or KIT mutations), reducing efficacy. Most importantly, most studies remain confined to cell/animal models, lacking clinical verification. Real-time monitoring of dynamic ubiquitination modifications (dose-dependent effects of USP15 stabilizing ACSL4) is impractical, obscuring the therapeutic window. Furthermore, future studies can focus the ubiquitin–proteasome system of GIST to predict the progression of disease as well as the level of resistance to imatinib while trying to intercept the resistance to better help patients to restrict this tumor.
